# Human placental extract attenuates neurological symptoms in the experimental autoimmune encephalomyelitis model of multiple sclerosis-a putative approach in MS disease?

**DOI:** 10.1186/s13317-020-00137-x

**Published:** 2020-10-04

**Authors:** Mir Hadi Jazayeri, Khadijeh barzaman, Reza Nedaeinia, Tayebe Aghaie, Morteza Motallebnezhad

**Affiliations:** 1 Department of Immunology, Faculty of Medicine, Iran University of Medical Sciences, Shahid Hemmat Highway, P.O Box: 14665-354, Tehran, 1449614535 Iran; 2 Immunology Research Center, Iran University of Medical Sciences, Tehran, Iran; 3 Pediatric Inherited Diseases Research Center, Research Institute for Primordial Prevention of Non-Communicable Disease, Isfahan University of Medical Sciences, Isfahan, Iran

**Keywords:** Human placental extract, IL-23, IL-27, Multiple sclerosis, EAE

## Abstract

**Background:**

Different studies have demonstrated the anti-inflammatory effects of human placental extract both in vivo and in vitro. Considering the chronic inflammatory nature of multiple sclerosis (MS) disease, we examined whether or not the administration of human placental extract is able to attenuate the neurological symptoms detected in experimental autoimmune encephalomyelitis (EAE) model of MS.

**Methods:**

The injected myelin oligodendrocyte glycoprotein (MOG) induced EAE in mice, and treatment began from day 4 post-injection by intraperitoneal administration of 0.2 mg/kg human placental extract, repeated every other day up to day 31 post-injection. At the end of the treatment, luxol fast blue (LBS) staining and hematoxylin and eosin (H&E) staining were performed to evaluate the demyelination of neurons and inflammatory responses, respectively. Further assessed were the serum concentrations of IL-23 and IL-27.

**Results:**

The administration of human placental extract was able to significantly reduce the mean clinical score in EAE mice, decrease the pro-inflammatory process and attenuate neural demyelination. Moreover, while the serum concentration of IL-23 was significantly diminished in the EAE mice receiving human placental extract compared to the non-treated EAE group, IL-27 concentration was significantly increased.

**Conclusions:**

Our findings demonstrated the administration of human placental extract could significantly attenuate the neurological symptoms in the EAE model of MS in part through modulating the serum levels of IL-23 and IL-27 and enhancing neuroprotection and myelin repair.

## Background

Characterized by the progressive demyelination of neurons, multiple sclerosis is the most prevalent chronic inflammatory disorder of the nervous system, affecting more than 2 million individuals worldwide [1]. Among different pro-inflammatory mediators identified in the central nervous system (CNS), those related to interleukin-12 (IL-12) cytokine family containing IL-12, IL-23, IL-35, and IL-27 have shown to be in close association with the etiology of MS. While the pro-inflammatory nature of IL-12 and IL-23 cytokines has been elucidated in several studies, additional anti-inflammatory properties have been attributed to IL-27. Consistently, it has been shown that the continuous administration of IL-27 in experimental autoimmune encephalomyelitis (EAE) model can significantly reduce the severity of the disease in animals [2–4]. Furthermore, knockout mice for IL-27 receptor or its downstream signaling mediators develop EAE disease with much more severe symptoms in comparison to normal mice [5, 6]. Based on translational studies performed on human, treating dendritic cells obtained from healthy donors, with IFN-β promotes secretion of IL-27 in vitro [7]. Similarly, ex vivo isolated plasmacytoid dendritic cells (*pDCs*) obtained from MS patients receiving IFN-β treatment for a period of one month, secreted higher amounts of IL-27 compared to the non-treated group [8]. Also, patients demonstrating good response to glatiramer acetate therapy also had elevated serum levels of IL-27 [9]. All these observations confirm the anti-inflammatory role of IL-27 in CNS autoimmunity, proposing it as a valuable target for treating MS. On the contrary, IL-23 exacerbates the demyelination of neurons through promoting the differentiation and maturation of Th17 cells. These cells are believed to be the key contributors in the development of encephalitogenic responses [10]. Therefore, any medication capable of increasing the serum concentration of IL-27, while decreasing IL-23, can be considered as a potential therapy for MS.

Human placenta is believed to encompass numerous biological molecules with a broad range of biological activities [11–13]. Different studies have shown that human placenta and its extract are able to modulate immune responses, protect and regenerate hepatocytes, regulate hormonal balance in women’s body, modulate monoamine oxidase activity in brain, and promote wound healing and the anti-coagulation process [14–19]. In line with these observations, different types of growth factors, their corresponding receptors, and other regulating molecules have been recognized in human placenta [20–24]. Currently, human placental extract is approved for clinical use to improve chronic liver dysfunction and alleviate menopausal symptoms in different countries [16, 25]. Along with these findings, the immuno-inhibitory function of human placental extract has been proven in numerous studies. For instance, it has been shown that the administration of placental extract in lymphocytes results in a cytostatic response and inhibition of responses to mitogenic stimuli [26]. In addition, by affecting T lymphocytes, placental extract is capable of inhibiting graft-versus-host disease and allogenic mixed lymphocyte reaction [27, 28]. Placental extract may further inhibit B cells activity because the secretion of antibody against immunized antigen is suppressed in the presence of placental extract [29]. Also, the suppressive effect of placental extract on inflammation caused by pro-inflammatory agents such as carrageenan is representative of its inhibitory effect on innate immune system [14]. Finally, from a clinical point of view, placental extract is applied in the treatment of pelvic inflammatory disease [30].

Based on such observations, we hypothesized that the immunomodulatory effects of human placental extract might be conducive to attenuating neurological symptoms in association with EAE model of MS. Therefore, in the current study, we demonstrated the protective impacts of human placental extract on the EAE model of MS. In addition, as Il-23 and IL-27 are the main modulators of inflammation in MS, the modulatory effects of the extract on these cytokines were also examined.

## Methods

### Animals

Adult female C57BL/6 mice (18–20 g in weight) were obtained from Pasteur institute of Iran (Tehran, Iran) and housed 4 per cage under a 12 h light/dark cycle at room temperature. Whole Experimental procedures was performed according to the NIH’s guidelines for the care and use of laboratory animals, which was approved by the Ethics Committee for Animal Research of Iran University of Medical Sciences, Tehran, Iran.

### Induction and treatment of EAE

EAE was induced by subcutaneous injection of 400 μg MOG_35–55_ peptide, emulsified in complete Freund’s adjuvant (Sigma Alderich) holding *Mycobacterium tuberculosis* with a concentration of 5 mg/ml to the symmetrical points in the left and right flanks of C57BL/6 mice on day zero. On the same day, mice were intra-peritoneally injected with 250 ng pertussis toxin (Sigma Alderich) dissolved in 200 μL saline (i.p.), repeated 48 h later. On a daily basis, the clinical symptoms associated with the disease were evaluated, and the mice were weighed, and scoring was performed according to the following established protocol: 0, no symptoms; 1, decreased or loss of tail tonicity; 2, flabby tail; 3, paralysis in one hind limb; 4, paralysis in both hind limbs; 5, paralysis of hind limb and the forelimb; and 6, death [31]. A facilitated route was also provided for the easy access of paralyzed mice to both water and food. At the beginning of the study, mice were randomly classified into three groups, each consisting of 5–6 mice which received the following treatments:

Group I (control group): mice in this group were all healthy (EAE was not induced) and only received vehicle (PBS) as treatment.

Group II (EAE negative control group): EAE was induced in these mice and treatment only consisted of PBS injection.

Group III (Human placental extract-treated EAE group): EAE was induced in these mice and treatment consisted of injecting 0.2 mg/kg human placental extract IP.

Treatments, either with PBS or Human placental extract, were administered every other day commencing from day 4 and ending on day 31. Body weight recording and EAE clinical scoring were also evaluated up to the end of day 31. On the following day, all mice were euthanized and spinal cord and brain tissues were collected for further histopathological analysis by luxol fast blue and H&E staining.

### Tissue preparation, luxol fast blue and H&E staining

Methods applied in this section of study are similar to those reported by Mozafari et al. [32]. Following anesthetization, mice were intracardially perfused with 0.1 M PBS, and then with 4% paraformaldehyde in 0.1 M PBS (pH 7.4). Thereafter, Lumbar spinal cords were collected and subjected to post-fixation overnight at 4 °C. The extent of demyelination was evaluated using luxol fast blue (LBS) staining while hematoxylin and eosin (H&E) staining was performed to the count the number of inflammatory cells and vacuolization.

For LBS, tissue samples were initially dehydrated in a series of alcohol concentrations and then cleared by incubating in xylene. Processed samples were then paraffin-embedded, blocked and sectioned into pieces of 5 μm in diameter utilizing rotary microtome (Lieca Microsystems, UK). Each section was rehydrated separately and stained for 3 h with 0.1% Luxol Fast Blue (British Drug House, UK) at 60 °C. Counterstaining was performed for 5 min using 0.1% Cresyl Fast Violet (Merck, Germany). Following the second step of dehydration and clearance with alcohol and xylene, sections were cover slipped and thoroughly screened for to identify demyelinated lesions using an Olympus BX-51 microscope. Upon the identification of a lesion site, 7 sections within the 40–50 μm interval were selected for quantitative analysis. Totally, 21 slides were evaluated in each group (n = 3) and myelin staining severity and extent of the demyelinated area were quantified utilizing ImageJ software (version 1.32j, NIH, USA). The extent of demyelination was reported as percentage of the total area. To calculate the myelination intensity, the myelin density stained inside the demyelinated area was normalized to the one observed in an intact region in the same section. It was performed following rehydration in alcohol concentration series and paraffin embedment. Staining was then performed with hematoxylin for 4 min and counterstaining was done with eosine for 1.5 min. Sections were then thoroughly washed, cover slipped and analyzed by an expert pathologist blinded to the identity of the groups. A total of 21 sections were obtained from each experimental group and analyzed by a pathologist to specify the number of presented inflammatory cells and vacuolization.

### Measurement of the serum levels of IL-23 and IL-27

At the end of the study (day +31), mice blood was collected by cardiac puncture and the collected sera were used to identify the application of the commercially available enzyme-linked immunosorbent assay (ELISA) related to the manufacturers protocol (Bioscience, UK).

### Statistical analysis

Data obtained from histological analysis, the serum concentrations of IL-23 and IL-27, and the total number of clinical scores in three groups were analyzed with the graph pad/Prism version 8 using one-way ANOVA, followed by Tukey–Kramer post-test. All experiments were represented in the form of Mean ± SD (standard deviation). Throughout the study, *p* values lower than 0.05 were regarded as statistically significant.

## Results

### Human placental extract treatment attenuated EAE

**Fig. 1:**
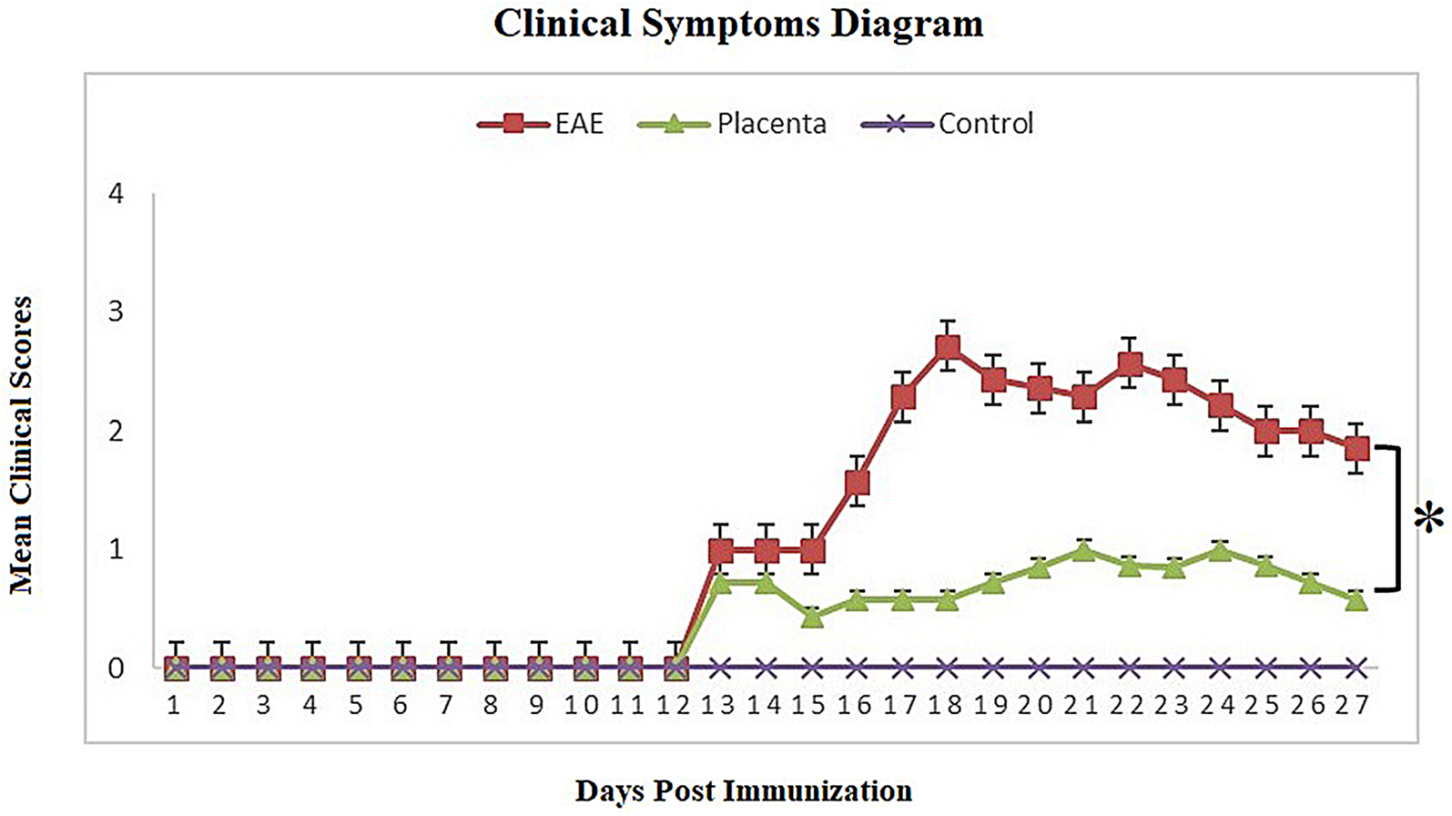
Evaluation of symptom severity scores in different groups showed that treatment with placenta extract significantly improved in this group (P = 0.0185)

**Fig. 2:**
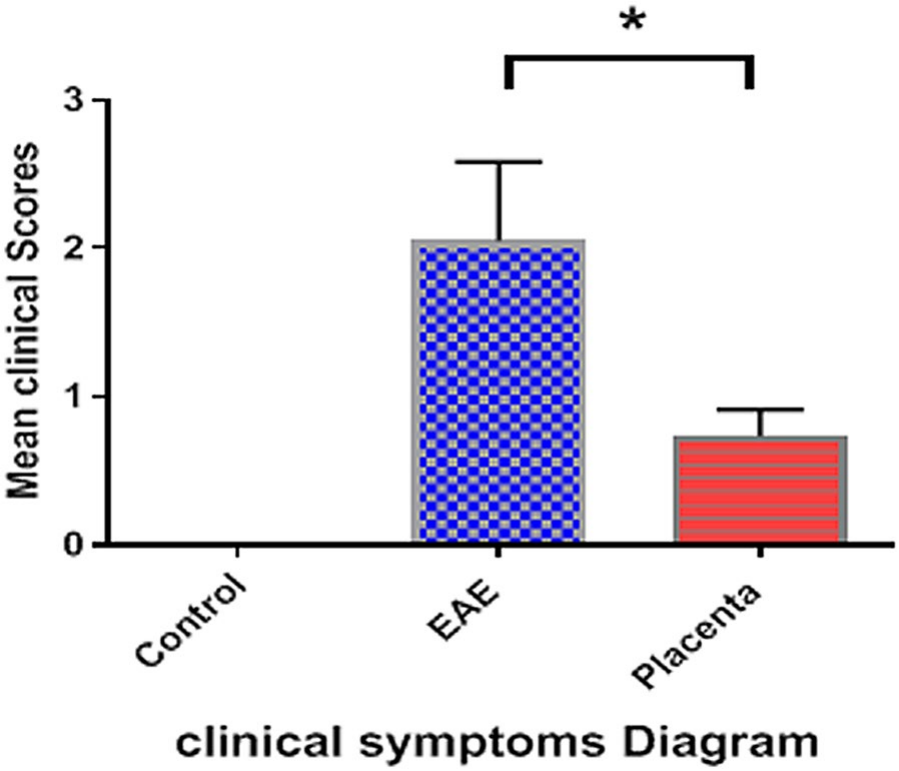
Diagram of clinical signs after human placenta extract administration. Treatment with human placental extract can significantly attenuate the severity of EAE symptoms

Figure 1 represents the clinical grade of EAE as a function of time for different groups. In both the control and human placental extract groups, clinical symptoms were commenced on day +12. However, the maximum mean clinical score (MMCS) of the treatment group receiving human placenta extract (1.32 ± 0.21) was significantly lower than the normal group (*P* < 0.0001), hence the conclusion that treatment with human placental extract can significantly attenuate the severity of EAE symptoms. Based on the histopathological studies performed on lumbar spinal cords isolated from the mice at the end of the study, a significantly reduced demyelination was observed following treatment with human placental extract compared to PBS-receiving group (Fig. 2). In addition, while the intensity of myelin was reduced in PBS treatment group, human placental extract was able to enhance myelin staining intensity.

### The effect of human placental extract on the body weight

**Fig. 3:**
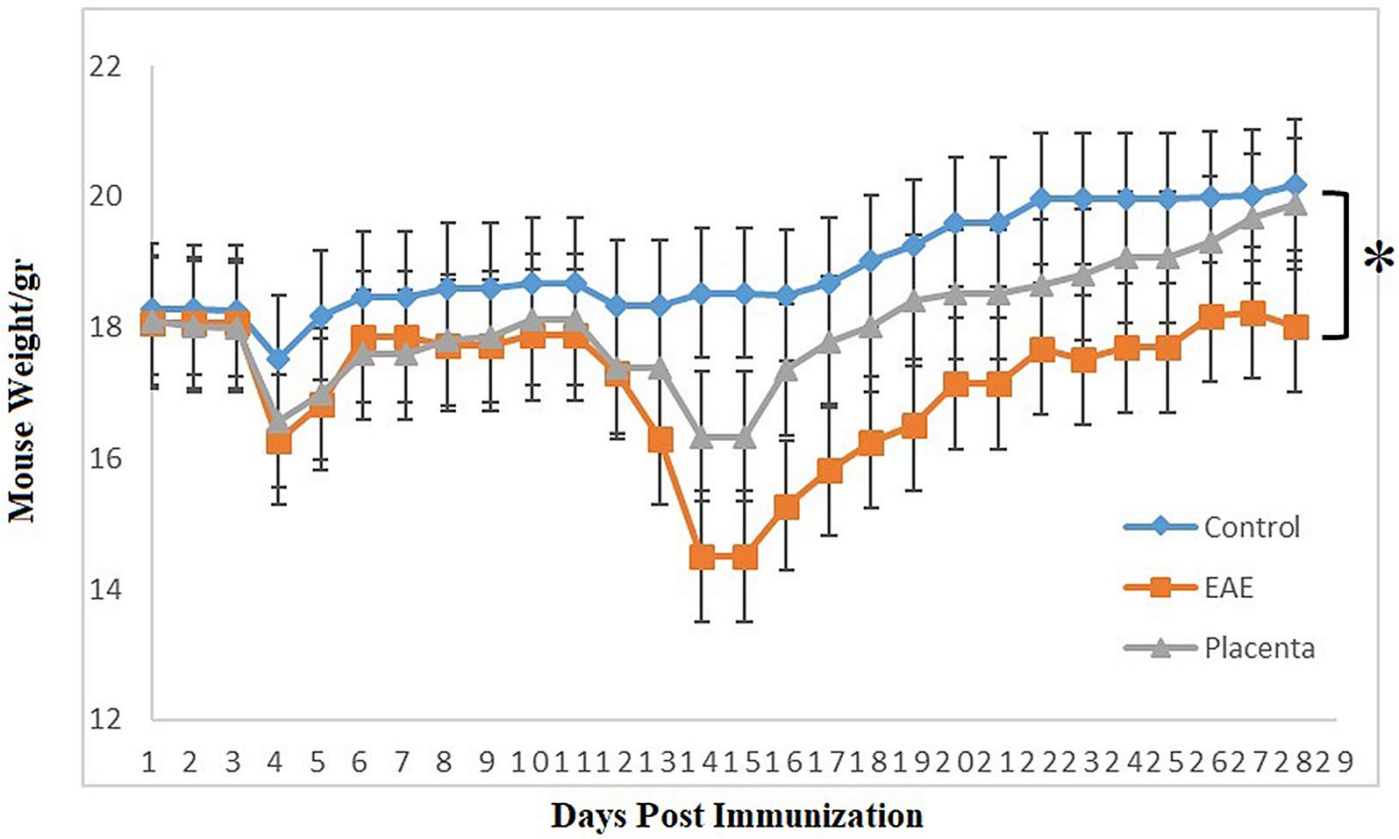
For statistical analysis of weight changes of mice after calculating the weight loss of each mice at the end of the period, statistical analysis of data was performed to compare groups weight loss with each other. There was a significant increase in weight changes in the treated group (p = 0.0222)

Figure 3 shows the effect of human placental extract on body weight of EAE mice in comparison to other groups including control negative and PBS receiving during the treatment period. In PBS receiving EAE mice, the mean body weight at the end of the treatment period (day 31) was significantly lower than the normal control group (P = 0.027). However, administration of human placental extract could significantly lower the trend of body weight loss in EAE mice; however, regarding this trend, no significant differences were observed between human placental extract receiving group and normal control group at the end of the treatment period. No significant toxicities related to the administration of human placental extract administration was observed throughout the survey period.

### The effect of human placental extract on leukocyte infiltration into CNS

**Fig. 4:**
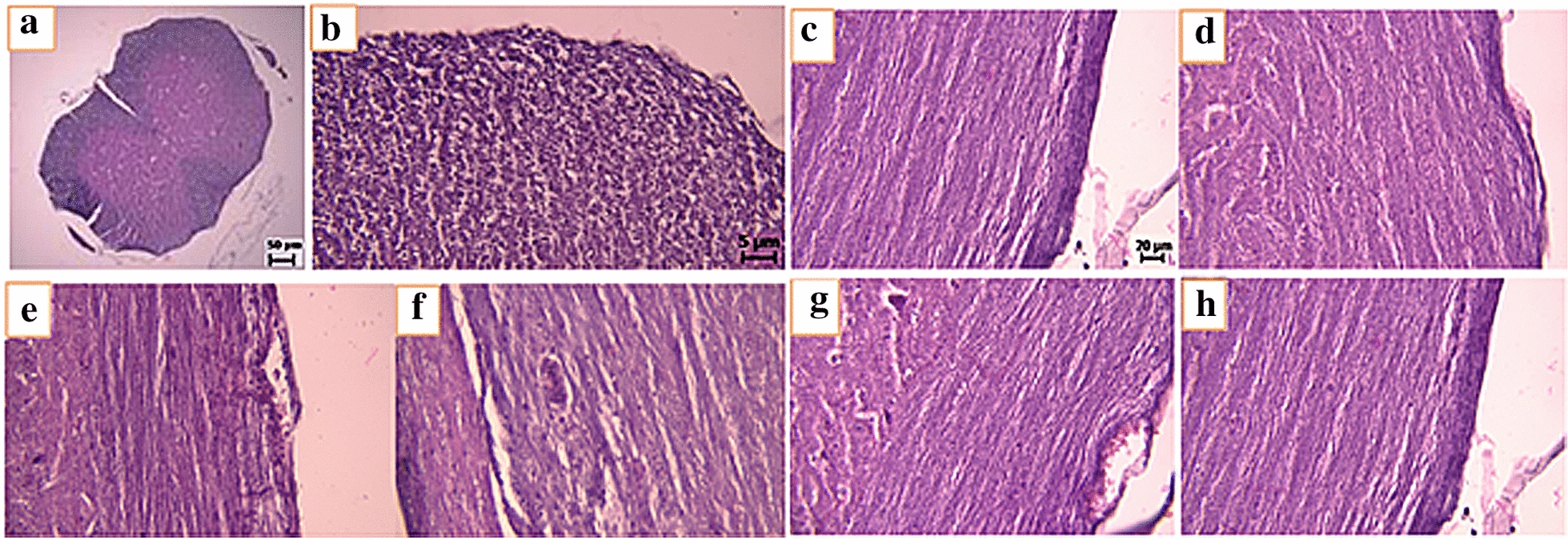
H&E staining converter; **a**, **b** of normal group (transverse section), **c, d** of normal group (longitudinal section), **e, f** of EAE group (longitudinal section), form **g** and **h** of treated group (longitudinal section). The presence of inflammatory areas and inflammatory cells infiltrated into the spinal cord tissue in the EAE group was observed in the EAE group compared to the normal group, with a decrease in inflammatory cells in the treated group

**Fig. 5:**
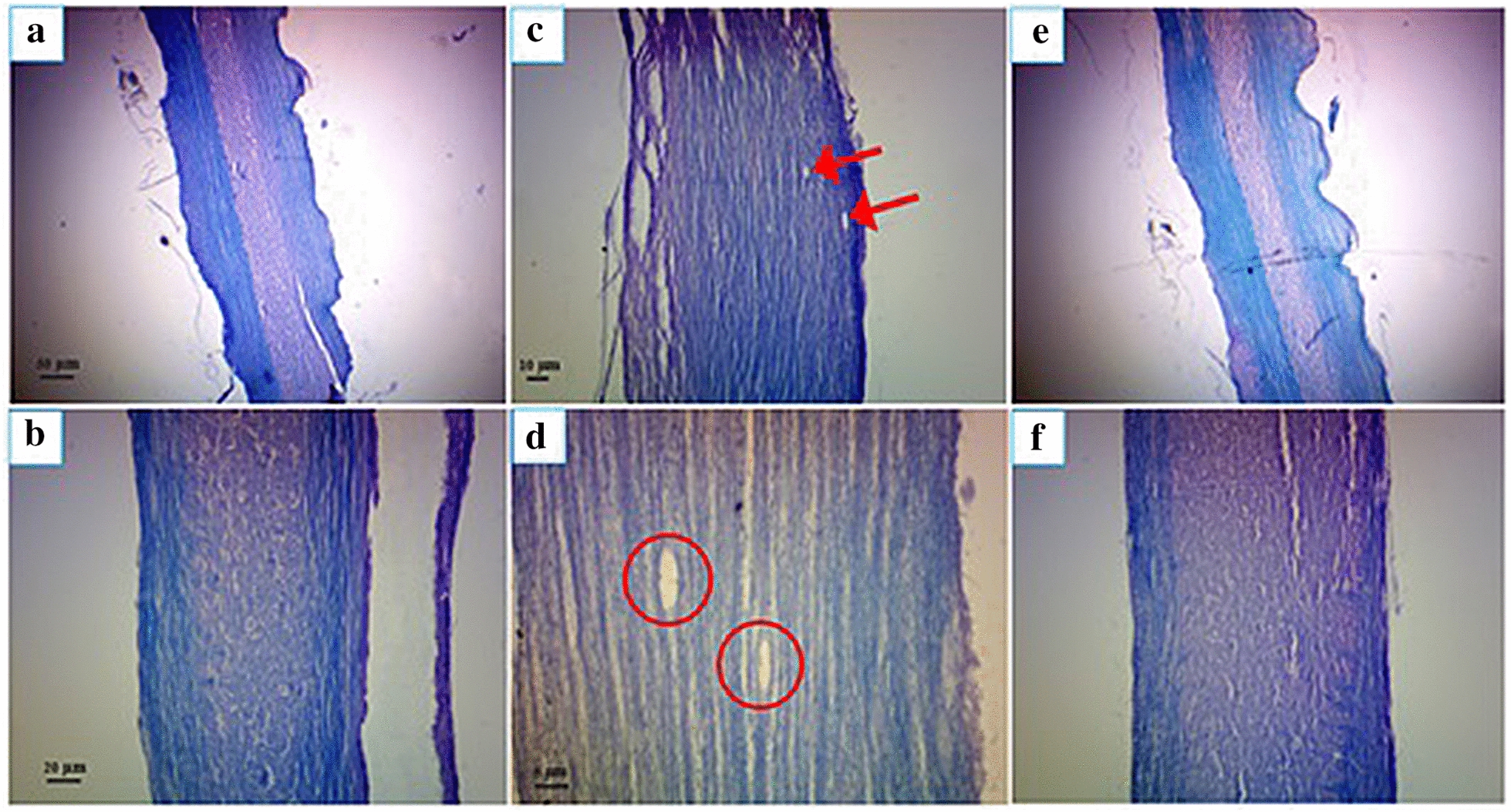
LFB staining of the longitudinal spinal cord. **a**, **b** There are no demyelination areas during the spinal cord of the healthy group. **c**, d Presence of demyelination areas during the spinal cord of the EAE group. **e**, **f** Significant reduction of the demyelinated areas during the spinal cord of the treatment group

Figure 4 shows the effect of human placental extract on leukocyte infiltration into CNS as compared with other groups, including control negative and PBS receiving groups at the end of the treatment period. According to Fig. 5, a severe CNS inflammation was observed in EAE mice receiving PBS compared to the normal control group. Interestingly, a significantly lower CNS inflammatory pattern was observed in mice treated with human placental extract. Therefore, it can be concluded that human placental extract can significantly inflammatory process in EAE mice.

### Effect of human placental extract on the expression profile of IL-23 and IL-27

**Fig. 6:**
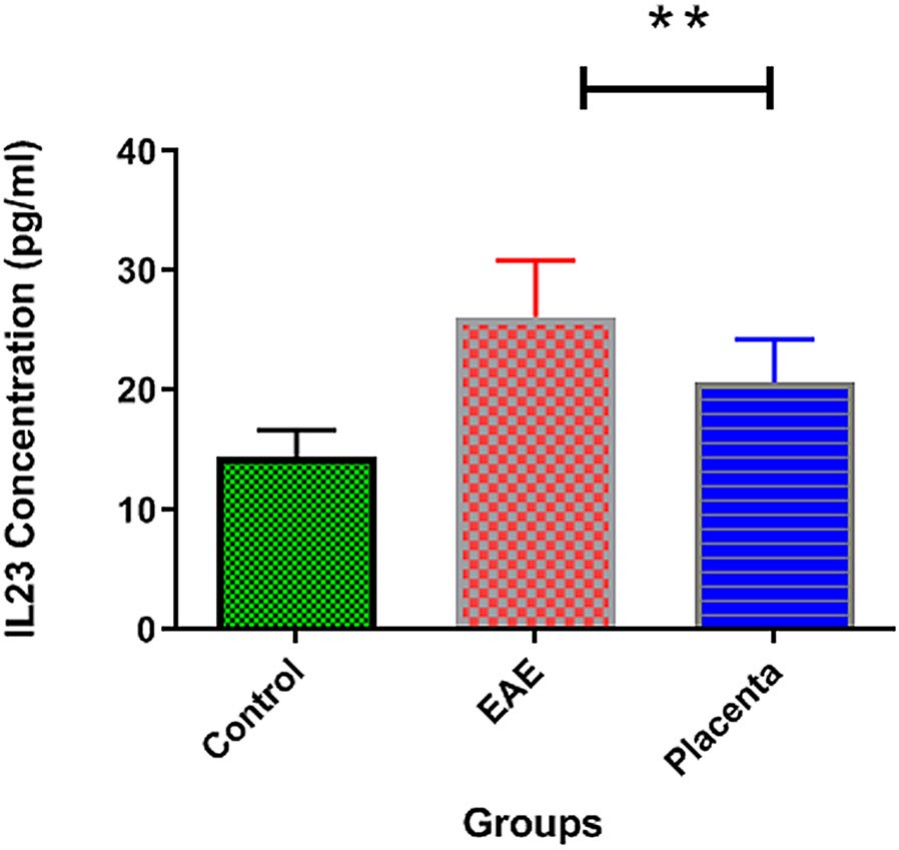
Diagram of IL-23 ELISA test results in placenta extract treated groups. IL-23 concentration was significantly decreased (p = 0.0036), compared to the EAE mice receiving PBS

**Fig. 7:**
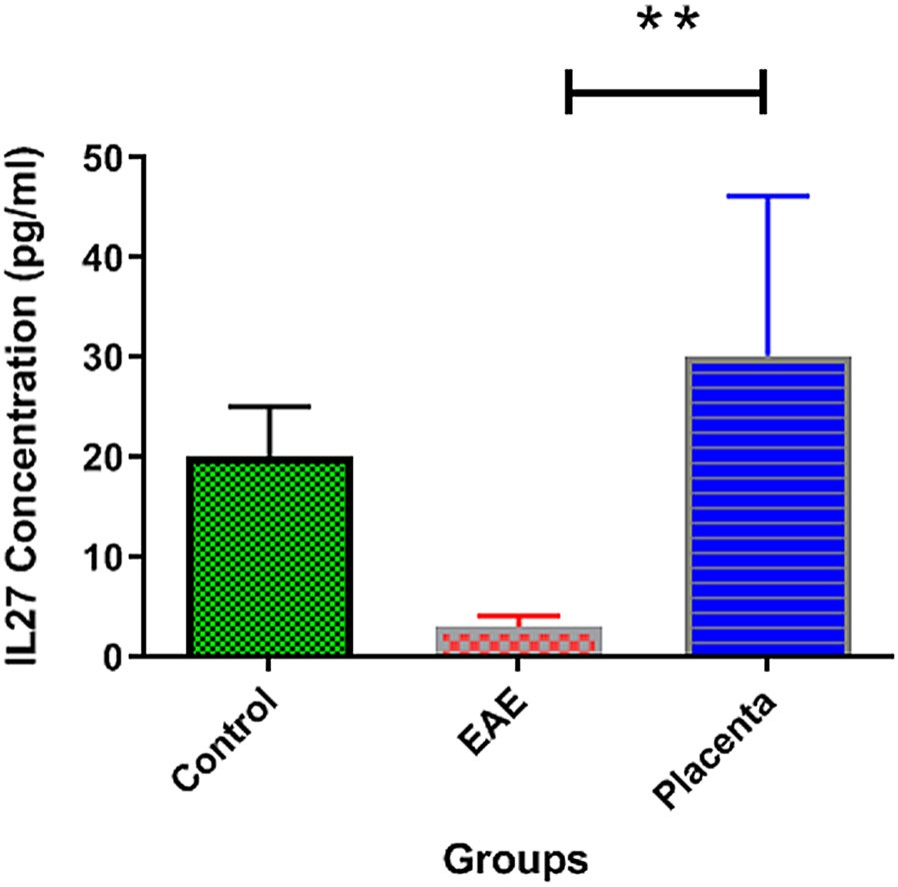
Diagram of IL-27 ELISA test results in placenta extract treated groups. Concentration of IL-27 was significantly increased (p = 0.0027) compared to the EAE mice receiving PBS

Figures 6 and 7 demonstrate the serum concentrations of IL-23 and IL-27 in normal control and EAE treated and non-treated with human placental extract. In the normal group, the mean plasma concentrations for IL-23 and IL-27 were 14.52 ± 2.126 and 20.3 ± 5.045, respectively. In EAE mice receiving PBS, the concentration of IL-23 was significantly increased (26.15 ± 4.747; p = 0.0036), while IL-27 was significantly reduced (3.12 ± 1.10; p = 0.0027). Although the administration of human placental extract was not able to restore the serum concentrations of IL-23 and IL-27 to the normal state, IL-23 concentration was significantly decreased (20.63 ± 3.645; p = 0.0036), while the concentration of IL-27 was significantly increased (30.18 ± 16.06; p = 0.0027) compared to the EAE mice receiving PBS. These results further corroborate the anti-inflammatory role of human placental extract in EAE mice.

## Discussion

Different studies have investigated the effects of various anti-inflammatory substances in the mouse models of multiple sclerosis [33, 34]. We also examined the anti-inflammatory effects of gold nanoparticles in a mouse model in a previous study, which revealed the anti-inflammatory effects of gold nanoparticles in the EAE mouse model [35, 36]. In the present research, the administration of human placental extract in EAE mice could improve clinical symptoms associated with EAE, in part attributable to the immunomodulatory effects of human placental extract on hyper activated immune system of EAE mice. The anti-inflammatory consequence of human placental extract has also been indicated in both in vitro and in vivo models of inflammation. For instance, treatment of LPS-stimulated RAW264.7 mouse macrophages with human placental extract resulted in a significant reduction in the section of TNF-α, IL-1β and IL-6 and other inflammatory mediators such as NO from these cells [37]. Therefore, the mechanism underlying the anti-inflammatory effects of human placental extract may partly be mediated through suppressing the expression of pro-inflammatory cytokines. Similarly, in the carrageenan-induced rat paw edema model of inflammation, administration of human placental extract could attenuate the swelling of rat’s paw [38]. Human placental extract is also able to inhibit granuloma formation in animal models of sub chronic inflammation. Finally, the administration of human placental extract in animal models of chronic inflammation, including rat AIA (Adjuvant-Induced Arthritis) model, could significantly attenuate inflammatory reactions [38]. The attenuation of neural demyelination evident in H&E and LBS stained sections, as well as the suppression of immune cells infiltration into the CNS further confirm the anti-inflammatory behavior of human placental extract in EAE mice. It has been shown that autoreactive CD4 + T-lymphocytes, especially Th17 cells, followed by Th1 and CD8 + , are the main players in the development of MS and the process of neural demyelination [39]. Therefore, human placental extract must be partly effective in attenuating destructive effects observed in these cells. The precise mechanism of the anti-inflammatory effects of human placental extract is yet to be fully elucidated; however, based on the foregoing observations, it can be concluded that: (A) human placental extract can partly affect NF-kB and inhibit the secretion of pro-inflammatory cytokines and mediators, and (B) human placental extract can attenuate the activity of T-cells because the demyelination of neurons mainly take place as a consequence of the destructive activity of these cells on neural cells myelin.

In parallel, perhaps the most important finding of our study was the modulatory effects of human placental extract on the serum levels of IL-23 and IL-27. As mentioned earlier, the serum level of IL-23 is usually higher and, in most cases, IL-27 is lower in MS patients compared with normal subjects, hence potential response modifiers for MS immunotherapy [40, 41]. For the first time, the present study showed that human placental extract was able to significantly increase the concentration of IL-27 (a cytokine which high concentration in the serum of MS patient results in good prognosis), and decrease IL-23 (a pro-inflammatory cytokine associated with poor prognosis in MS patients). Thus, another protective effect of human placental extract against EAE may be attributed to the modulation of the balance between IL-23 and IL-27 serum concentrations. Given the ability of IL-23 to promote the secretion of IL-10 and regulate IL-17 production by CD4 + T cells [42], one may propose that the administration of human placental extract in the first place, may suppress the destructive activity of autoreactive T cells in EAE. Similarly, human placental extract can suppress the secretion of several pro-inflammatory mediators, including IL-23 and several other mediators mentioned in different studies, resulting in the attenuation of inflammation and the mean clinical score in EAE mice [43–45].

## Conclusions

Although, more evaluations are needed, the results of the current study display the protective effects of human placental extract in the EAE model of MS which may partly be mediated through the downregulation of the serum levels of IL-23, upregulation of IL-27. Thus, human placental extract may be considered as a putative therapeutic agent for the management of clinical features observed in MS.

## Data Availability

The datasets used and/or analysed during the current study are available from the corresponding author on reasonable request

## Abbreviations

MS: Multiple sclerosis
EAE: Experimental autoimmune encephalomyelitis
MOG: Oligodendrocyte glycoprotein
LBS: Luxol fast blue
H&E: Hematoxylin and eosin
CNS: Central nervous system
*pDCs*: Plasmacytoid dendritic cells
ELISA: Enzyme-linked immunosorbent assay
MMCS: Maximum mean clinical score
AIA: Adjuvant induced arthritis
