# Correction to: Human placental extract attenuates neurological symptoms in the experimental autoimmune encephalomyelitis model of multiple sclerosis-a putative approach in MS disease?

**DOI:** 10.1007/s12026-022-09289-6

**Published:** 2022-08-11

**Authors:** Mir Hadi Jazayeri, Khadijeh barzaman, Reza Nedaeinia, Tayebe Aghaie, Morteza Motallebnezhad

**Affiliations:** 1 Department of Immunology, Faculty of Medicine, Iran University of Medical Sciences, Shahid Hemmat Highway, P.O Box: 14665-354, Tehran, 1449614535 Iran; 2 Immunology Research Center, Iran University of Medical Sciences, Tehran, Iran; 3 Pediatric Inherited Diseases Research Center, Research Institute for Primordial Prevention of Non-Communicable Disease, Isfahan University of Medical Sciences, Isfahan, Iran

**Correction to: Autoimmun Highlights (2020) 11:14**

https://doi.org/10.1186/s13317-020-00137-x

In this article, the author would like to correct a typo in the writing of 0.2 mg/kg instead of 20 mg/kg in the introduction and body of the thesis.

The original article has been corrected.

